# Different Effects of Startling Acoustic Stimuli (SAS) on TMS-Induced Responses at Rest and during Sustained Voluntary Contraction

**DOI:** 10.3389/fnhum.2016.00396

**Published:** 2016-08-05

**Authors:** Yen-Ting Chen, Shengai Li, Ping Zhou, Sheng Li

**Affiliations:** ^1^Department of Physical Medicine and Rehabilitation, University of Texas Health Science CenterHouston, TX, USA; ^2^The Institute for Rehabilitation and Research Memorial Hermann Research Center, The Institute for Rehabilitation and Research Memorial Hermann HospitalHouston, TX, USA; ^3^Guangdong Work Injury Rehabilitation CenterGuangzhou, China

**Keywords:** startling acoustic stimulus, isometric contraction, transcranial magnetic stimulus, motor evoked potential, silent period

## Abstract

Previous studies have shown that a habituated startling acoustic stimulus (SAS) can cause a transient suppression of motor evoked potentials (MEPs) induced by transcranial magnetic stimulation (TMS) during light muscle contraction. However, it is still unknown whether this phenomenon persists when at rest or during a sustained voluntary contraction task. Therefore, the purpose of this study was to determine whether a conditioning SAS has different effects. TMS was delivered to the hot spot for the left biceps on 11 subjects at rest both with and without a conditioning SAS. Of the 11subjects, 9 also had TMS delivered during isometric flexion of the left elbow, also with and without a conditioning SAS. TMS-induced MEPs, TMS-induced force, and silent periods were used to determine the effect of conditioning SAS. Consistent with previous findings, TMS-induced MEPs were smaller with a conditioning SAS (0.49 ± 0.37 mV) as compared without the SAS (0.69 ± 0.52 mV) at rest. However, a conditioning SAS during the voluntary contraction tasks resulted in a significant shortening of the MEP silent period (187.22 ± 22.99 ms with SAS vs. 200.56 ± 29.71 ms without SAS) without any changes in the amplitude of the MEP (1.37 ± 0.9 mV with SAS V.S. 1.32 ± 0.92 mV without SAS) or the TMS-induced force (3.11 ± 2.03 N-m with SAS V.S. 3.62 ± 1.33 N-m without SAS). Our results provide novel evidence that a conditioning SAS has different effects on the excitability of the motor cortex when at rest or during sustained voluntary contractions.

## Introduction

An acoustic startle reflex (ASR) is the involuntary motor activation in response to a loud and unexpected auditory stimuli (Valls-Solé, 2012). ASRs are mediated by a relatively simple neuronal circuit located in the lower brainstem. The proposed circuit of the ASR in humans involves the cochlear nucleus, the caudal pontine reticular nuclei, and the motoneurons of the brainstem and the spinal cord activated through the medial reticulospinal (RS) tract (Davis et al., 1982; Lee et al., 1996; Koch, 1999). Acoustic startle reflexes are easily habituated in humans. Only a few overt startle reflex responses may be elicited in succession and such responses may vary in amplitude between trials (Brown et al., 1991). Even after habituation to the ASR, ensuing startling acoustic stimuli (SAS) can still activate the reticular system non-reflexively (Kühn et al., 2004). In healthy subjects, it has been shown that a SAS delivered 50 ms prior to TMS delivery decreases the amplitudes of the TMS-induced motor evoked potentials (MEP) in both arm and leg muscles (Furubayashi et al., 2000; Fisher et al., 2004; Kühn et al., 2004; Ilic et al., 2011). When the same SAS conditioning is given to the same subjects prior to subcortical electrical stimulation (SES), facilitation of the MEPs in the same muscles is observed. Since SES bypasses the motor cortex, it has been argued that a SAS activates the reticular system via polysynaptic reticulo-cortical projections, which transiently inhibit the motor cortex, resulting in SAS-induced MEP inhibition, i.e., cortical inhibition (Kühn et al., 2004). In contrast, a SAS imposes an opposite effect on the spinal motor system, possibly mediated by stimulation of reticulospinal pathways. Concomitant with cortical inhibition, spinal motor neuron excitability is enhanced with a conditioning SAS delivered prior to TMS as shown by increased H-reflex amplitudes (Ilic et al., 2011).

The above SAS-preconditioned TMS experiments were performed while subjects maintained a small, unquantified amount of muscle activity. It is unknown whether the effect of a SAS on the excitability of the cortical and spinal motor systems is different between resting and sustained voluntary contraction conditions. This is important whether SAS could be integrated into a stroke motor rehabilitation program. Animal studies have demonstrated that the reticulospinal system is the major descending system to compensate for losses in the corticospinal tract following stroke (Nathan and Smith, 1955; Lemon, 2008; Sakai et al., 2009; Riddle and Baker, 2010; Ortiz-Rosario et al., 2014). The RS system has been shown to facilitate movement initiation and coordination in both healthy (Valls-Solé et al., 1999; Anzak et al., 2011a; Fernandez-Del-Olmo et al., 2014) and stroke (Honeycutt et al., 2013) subjects. Acoustic stimuli have been integrated into therapies for initiating and pacing voluntary movement such as in music therapy or auditory cueing (Whitall et al., 2000; Schneider et al., 2007; Jun et al., 2013; Pollock et al., 2014), possibly via stimulation of the RS system. It is reported that SAS could potentially be used to enhance one's maximal voluntary strength in healthy subjects (Anzak et al., 2011a) and in patients with Parkinson's disease (Anzak et al., 2011b). It is important to know whether SAS could improve force output in patients who usually undergo therapy and exercise repetitively at submaximal levels.

It has been demonstrated that a conditioning SAS reduces MEP when low muscle activity is maintained (Furubayashi et al., 2000; Fisher et al., 2004; Kühn et al., 2004; Ilic et al., 2011). However, the level of muscle activation was vaguely defined in these studies, such as “slightly pre-innervated” or “slight constant voluntary contraction.” It remains unclear whether this light pre-contraction could alter the SAS-induced cortical inhibition. Accordingly, the primary goal of this study was to examine the effect of a conditioning SAS on the motor system at rest as well as during precisely defined voluntary contractions. A well-established SAS-TMS paradigm (Furubayashi et al., 2000; Fisher et al., 2004; Kühn et al., 2004; Ilic et al., 2011) was used in this study. Healthy subjects were recruited and instructed to perform a sustained elbow flexion at a defined level or at rest. We hypothesized that the conditioning SAS could have different effects on the motor cortex and the descending corticospinal projections during both conditions of rest and of sustained contractions.

## Methods

### Participants

Eleven healthy adults (Age: 31.18 ± 6.18 years; 2 women) participated in this study. All subjects reported being healthy without any known neuromusculoskeletal impairments and were right-handed. The Committee for the Protection of Human Subjects at the University of Texas Health Science Center at Houston approved the procedures of this study. All participants provided written informed consent before participating in the study.

### Experimental setting

Each subject was seated comfortably in an upright position with the left arm in a customized arm device with the following configuration. The left shoulder joint was placed approximately in 45° of flexion and 30° of abduction, while the elbow was flexed to 90°. The left forearm was secured against two adjustable metal plates with a padded strap ~2–4 inches proximal from the wrist. The left forearm was kept in a neutrally rotated position. The right arm was rested symmetrically on an adjustable height table. A 20 inch monitor (Model: 2001FP, Dell Computer Corp., Texas, USA) was located about 1 meter in front of the subject at eye level. The monitor was used to display the force produced by the elbow flexion using a custom-written program in LabView® (National Instrument™ Inc., Austin, Texas, USA). All subjects affirmed that they could see the display clearly. A loud startle sound (Microsoft system warning sound, 1 KHz tone of 50 ms) was generated by the computer through a sound card (Model: Sound Blaster Extreme, Creative Technology Ltd.) and a speaker (Model: HS50M, YAHAMA Corp., Hamamatsu, Japan) at 100 dB. The speaker was located 30 cm behind the subject, at ear level.

Elbow flexion force was measured using a torque sensor (Model: TRS-500, Transducer Techniques, Temecula, CA, USA). The sensor was located in line with the center of the rotation of the left elbow joint. The elbow flexion torque signal was sampled at 1000 Hz with a NI-DAQ card (Model: PCI-6229, National Instruments, Austin, TX, USA). Surface EMG electrodes (Delsys Inc., Boston, MA, USA) were placed on the biceps muscles bilaterally according to the European Recommendations for Surface Electromyography (Hermens et al., 2000). The EMG signals were band pass filtered from 20 to 450 Hz, amplified 1000 times, and then sampled at 1000 Hz using the same NI-DAQ card. Both EMG and force signals were stored on a personal computer.

### Experimental tasks

In this study, we aimed to examine the effects of a habituated startling acoustic stimulation (SAS) on TMS-induced responses from the left biceps brachii muscle at rest and during muscle contraction. There were four tasks as follows: (1) TMS at rest without SAS (REST_TMS_); (2) TMS at rest with a conditioning SAS (REST_SAS-TMS_); (3) TMS during voluntary muscle contractions without SAS (VOLT_TMS_); (4) TMS during voluntary muscle contractions with a conditioning SAS (VOLT_SAS-TMS_). Prior to the main experiments, the TMS hotspot for the left biceps muscle was localized and maximum voluntary contraction (MVC) force of left biceps muscle was estimated.

To find the hotspot for left biceps for each subject, a single-pulse TMS stimuli (BiStim2, Magstim Corp., UK) was set at an intensity of 75% of the maximum stimulator output (equal to 84.75% maximum stimulator output on Magstim 200^2^, Magstim Corp., UK) while subjects held their left forearm off the table in about 90° of elbow flexion (lower than 10% of MVC). TMS was delivered over the right primary motor cortex using a figure-of-8 shaped stimulation coil (a 35-mm mean diameter of each wing, Model: BiStim^2^, MagStim Corp., UK). The hotspot was defined where the largest increment in elbow flexion was produced in three consecutive trials. We marked the spot on the scalp with a gel ink pen for the rest of the tasks. We used 75% of maximum stimulator output for all the subjects and tasks in this study because we intended to additionally elicit EMG responses ipsilateral (iMEP) to TMS delivery (Harris-Love et al., 2011). However, we were unable to elicit the iMEP with this experimental setup at our level of voluntary contraction. For this reason, we have not reported any iMEP results in this study.

MVC force was estimated 3 times for left elbow flexion. The subjects were asked to produce a maximum elbow flexion force for 3–5 s. The highest force among 3 attempts was considered the MVC force to predefine the target force in the main experiment. 1-min of rest was provided between consecutive MVC attempts. Following the MVC task, 8 consecutive SAS were delivered at 100 dB to ensure habituation of the startle reflex (Kühn et al., 2004).

There were two main experimental conditions.

**Figure 1:**
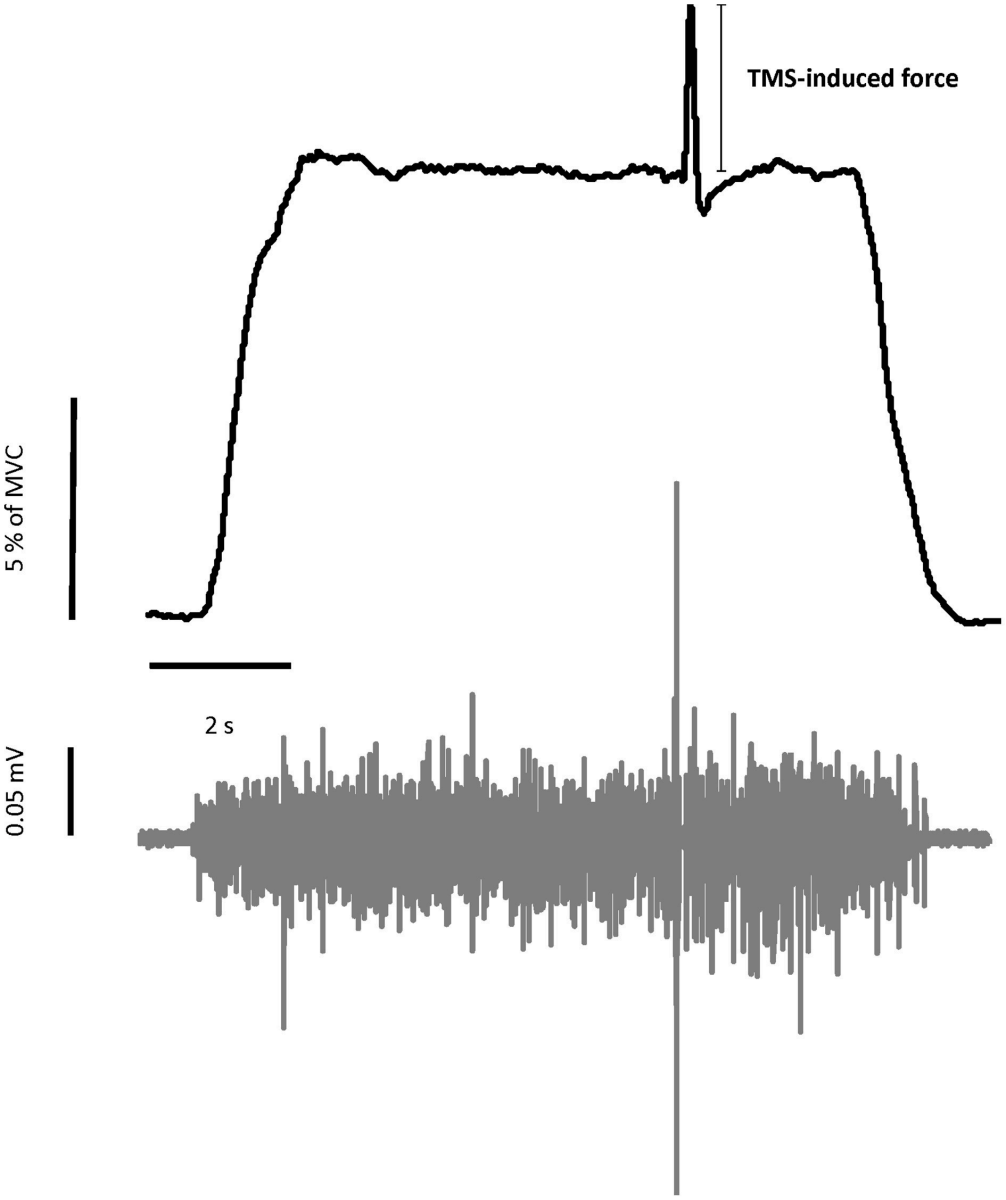
**Representative trial of force and EMG signals during voluntary contraction tasks**. Upper figure showed the force trajectory of left elbow flexion. TMS-induced force was quantified as the difference between the background force and the peak force elicited by TMS. The bottom figure showed the raw EMG signal of left biceps muscle. MEP and silent period can be seen in the raw EMG signal.

1. TMS at rest, with or without a conditioning SAS: The subjects were asked to relax. When there was no conditioning SAS (REST_TMS_), TMS was delivered to the hotspot of the right motor cortex randomly between 7 and 11 s during a 12-s trial. In the trials with a conditioning SAS (REST_SAS-TMS_), a 100 dB SAS was programmed to be delivered 50 ms prior to the randomized delivery of TMS. Force signals were not collected in these conditions.
2. TMS during voluntary muscle contractions, with or without a conditioning SAS: Before a trial began, a target force level of 10% of the MVC was provided as a red horizontal line in the middle of the monitor. The real-time force signal was provided as a white trace on the screen. It ran from left to right during each 12-s trial. For each trial, the subjects were asked to wait 1 s and then increase their left elbow isometric contraction force to reach the target within 2 s. Subjects were encouraged to match the white line (force) with the red line (target) as closely as possible throughout the trial. In VOLT_TMS_ tasks, TMS was delivered to the hotspot on the right motor cortex randomly between 7 and 11 s. During the task with a conditioning SAS (VOLT_SAS-TMS_), SAS was programmed to be delivered 50 ms prior to the randomized TMS delivery. One to three practice trials were given to the subjects for familiarization of the force task. Figure 1 illustrated the raw data of a representative VOLT task.

All 11 subjects participated in the REST tasks. Only 9 of them performed the VOLT tasks. At the end of each trial of the above tasks, a graph of TMS-induced responses in an 80 ms window (30 ms prior and 50 ms after the TMS delivery) was available for review. Any trial with potential outlier responses, e.g., a sudden change in MEP amplitude or noisy response, was discarded. The hotspot was then verified periodically throughout the experiments. Each task had six trials with excellent TMS-induced responses. Adequate rest breaks were allowed between trials to minimize any possible fatigue effect.

### Data analysis

**Figure 2:**
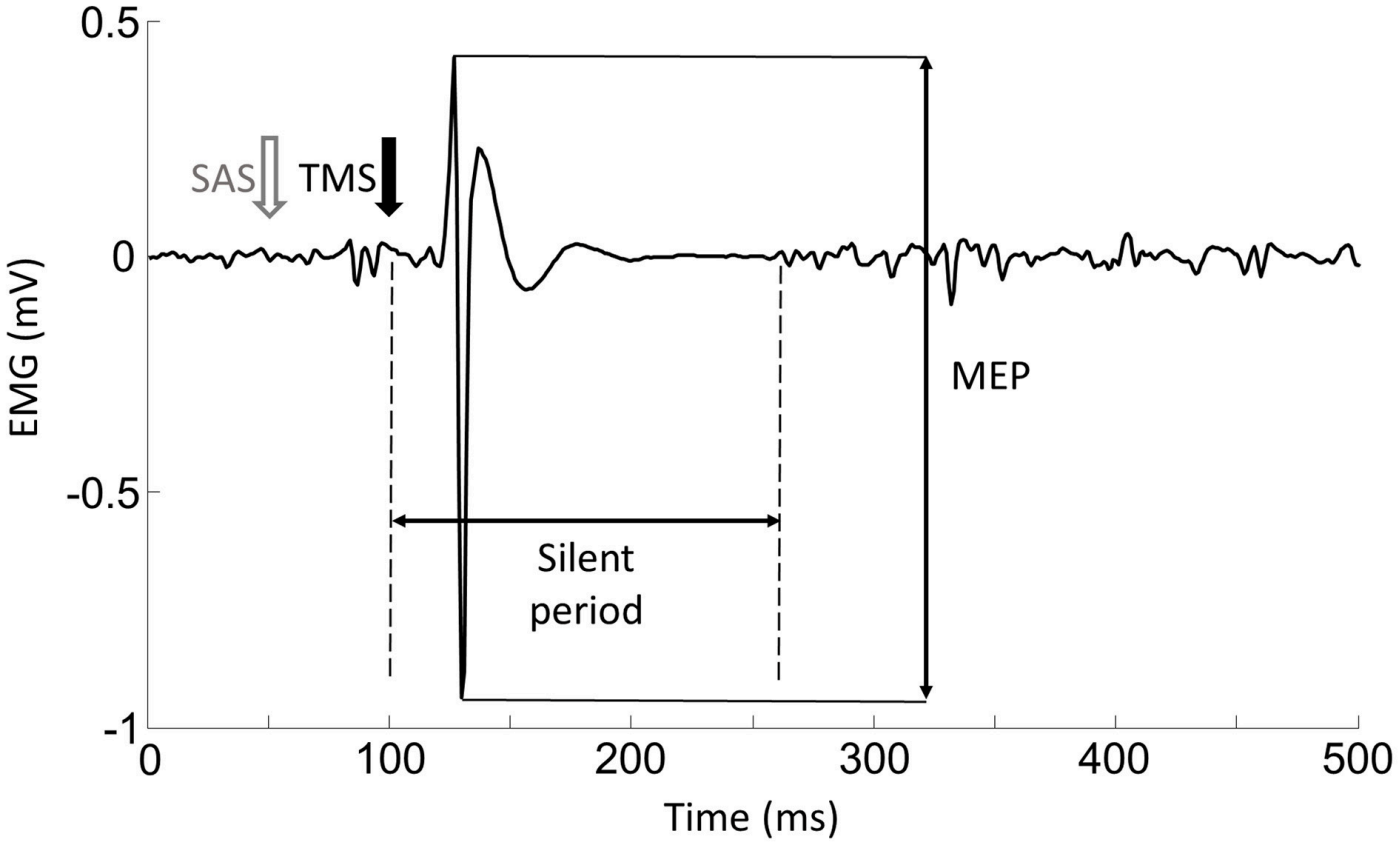
**Representative trial of MEP and silent period**.

Data was analyzed off-line using custom-written Matlab® programs (Math Works™ Inc., Natick, Massachusetts, USA). We extracted the force and EMG signals 100 ms before and 400 ms after the onset of the TMS (a window of 500 ms) from the raw data. The raw EMG signal was detrended in order to remove the offset and high-pass filtered at 5 Hz with a fourth-order, zero-lag Butterworth digital filter before further analysis for all measured parameters except for the silent period calculation. Similar to our recent analysis methods (Park and Li, 2013), the following parameters were calculated in this study:

1. **Motor Evoke Potentials (MEPs):** As shown in Figure 2, the EMG response occurred shortly after the TMS delivery, and was followed by a silent period. (1) Background EMG was defined as the mean of the rectified EMG calculated over the 100-ms window prior to the TMS delivery for each trial. (2) MEP latency: MEP latency was quantified as the time between the TMS delivery to the time point when the EMG signal exceeded 2 standard deviations of the EMG in the aforementioned pre-TMS 100-ms window for each trial (Wiethoff et al., 2014). (3) MEP amplitude: To calculate the MEP amplitude, we quantified the peak-to-peak EMG amplitude of the MEP with the time window from the MEP onset to 50 ms after the TMS delivery. (4) Silent period: before calculating the silent period, the EMG signals were (1) high-passed using a fourth-order, zero-lag Butterworth digital filter with a cut-off frequency of 65 Hz in order to remove slow oscillations (keep the signals related to the muscle action potential, Neto and Christou, 2010) and 60 Hz noise; (2) rectified; (3) low-passed using a fourth-order, zero-lag Butterworth digital filter with a cut-off frequency of 30 Hz to smooth the EMG signals. In order to find a clear silent period, the EMG signals were averaged from all the trials for each task and each subject. As demonstrated in **Figure 5A**, The silent period was quantified as the time between the onset of the MEP to the time when the EMG signal reached the background EMG level again (Kojima et al., 2013).
2. **TMS-Induced Force:** Background force was calculated as the mean force over a 100-ms window prior to the TMS delivery. TMS-induced force increment was quantified as the difference between the background force and the peak force elicited by TMS during the VOLT_SAS-TMS_ and VOLT_TMS_ tasks.

### Statistical analysis

The major dependent variables were: (1) background EMG; (2) MEP latency; (3) MEP amplitude; (4) silent period; (5) background force; (6) TMS-induced force. Two-way repeated measure ANOVAs were used to compare the effect of SAS on TMS-induced MEP responses, with factors of CONTRACTION (rest or 10% MVC) and SAS (with and without). Paired *t*-tests were used to test the effect of SAS on the induced force. The alpha level for all statistical tests was 0.05. Data is reported as mean ± SD within the text and as mean ± SEM in the figures. Only the significant main effects are presented, unless otherwise noted.

## Results

### MVC and background EMG and force

The average MVC force for all the subjects was 37.92 ± 6.91 Nm. The background force was not significantly different between the VOLT_SAS-TMS_ task (3.89 ± 0.76 Nm) and the VOLT_TMS_ task (3.88 ± 0.79 Nm). The background EMG was not significantly different (*p* > 0.05) between the REST_SAS-TMS_ task (0.0033 ± 0.0026 mV) and the REST_TMS_ task (0.0035 ± 0.0014 mV). The background EMG was also not significantly different between the VOLT_SAS-TMS_ task with (0.0095 ± 0.0065 mV) and the VOLT_TMS_ task (0.0095 ± 0.0072 mV).

### The effect of SAS

**Figure 3:**
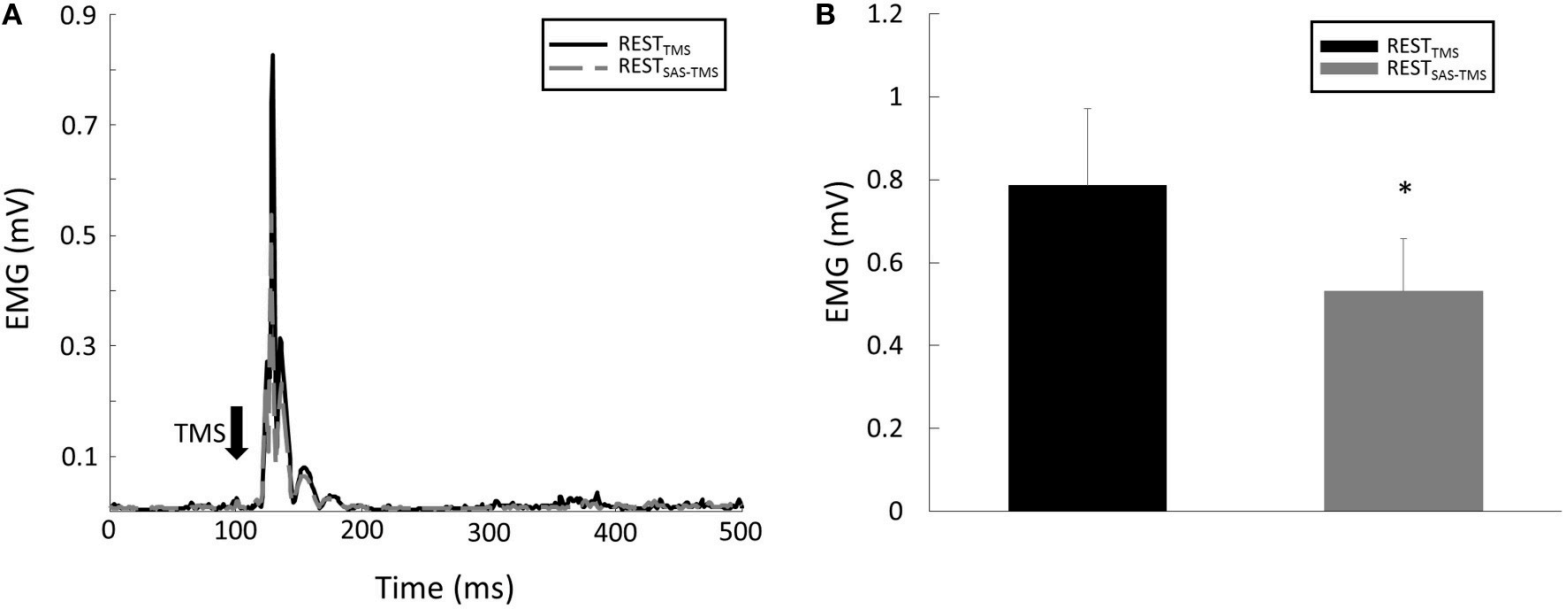
**(A)** Representative trials of MEP responses during the REST tasks. Note: the EMG signals were rectified in this figure and in the following representative figures. **(B)** The average MEP amplitude was smaller during the REST_SAS-TMS_ task compared with the REST_TMS_ task. ^*^Indicates significant difference (*p* < 0.05).

**Figure 4:**
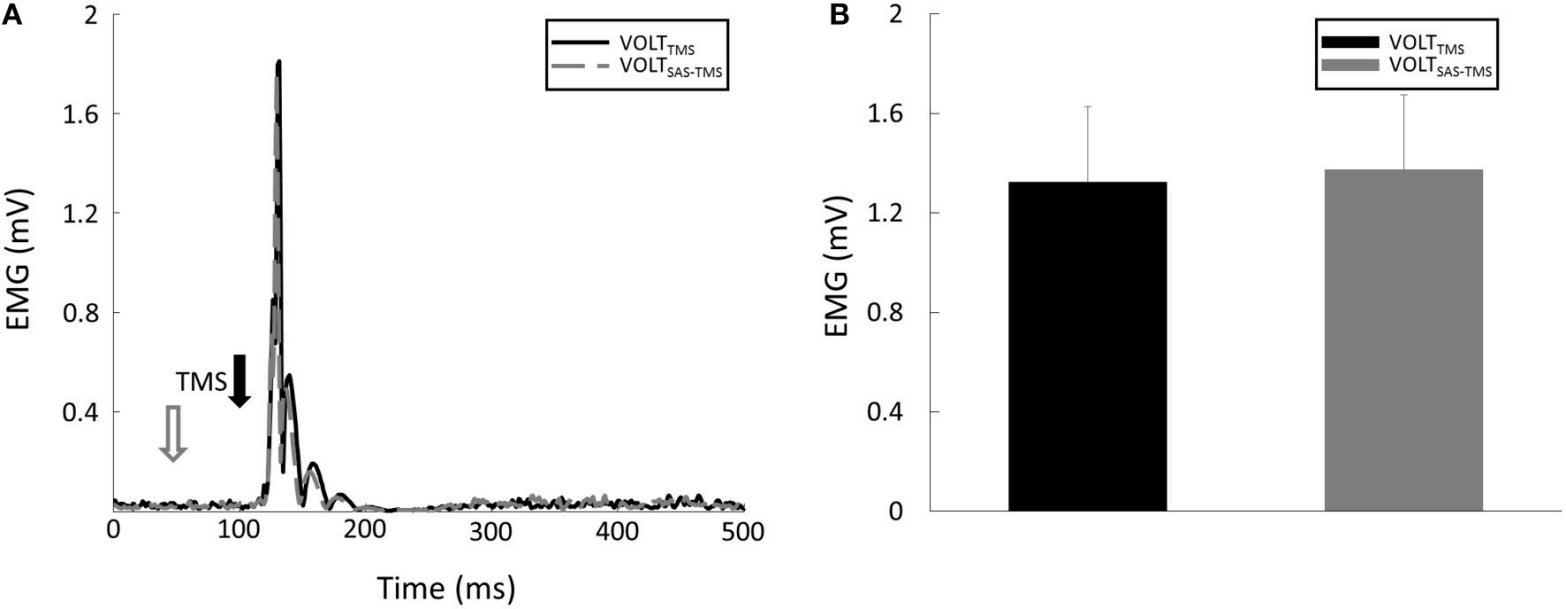
**(A)** Representative trials of MEP responses during VOLT tasks. **(B)** Averaged MEP amplitudes were similar between VOLT_SAS-TMS_ and VOLT_TMS_ tasks.

**Figure 5:**
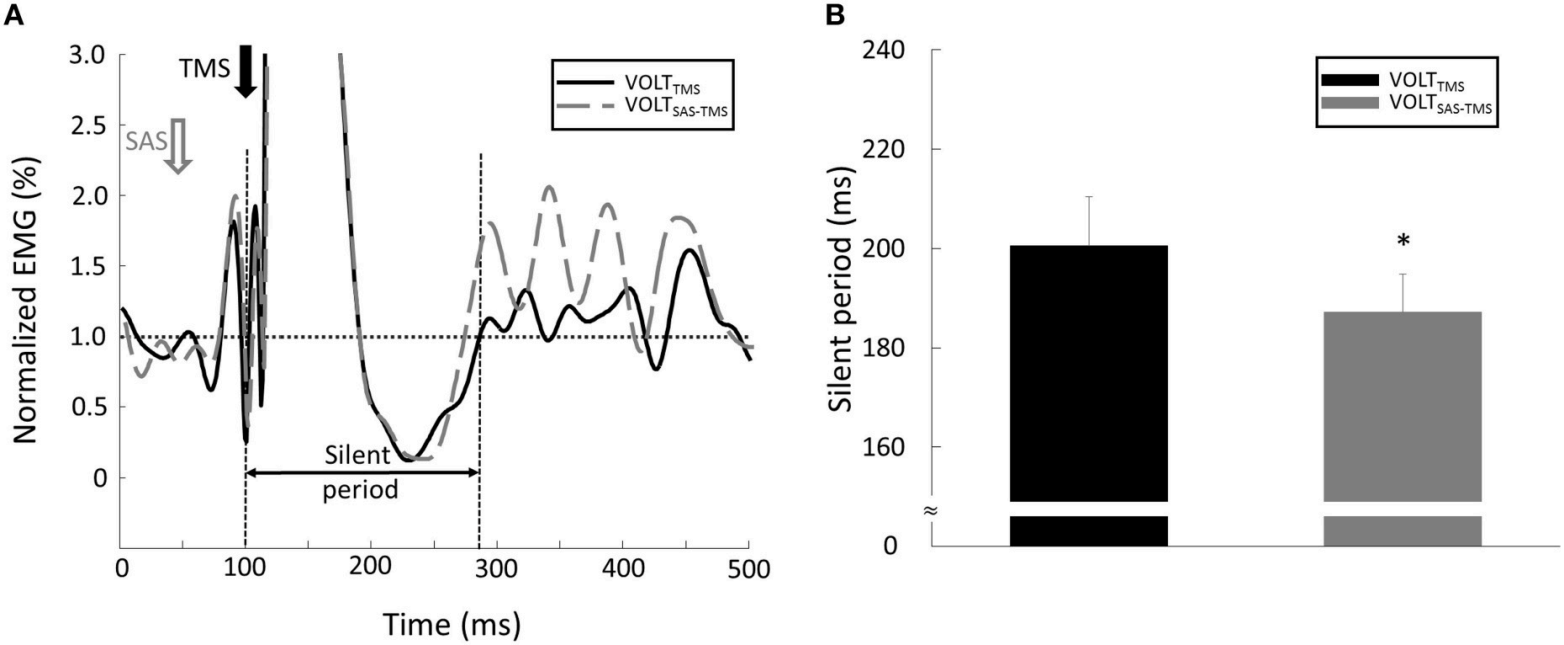
**(A)** Representative trial of silent period during VOLT tasks. Note: in order to clearly compare the signals between different tasks, the EMG signal was normalized by the background EMG amplitude in this figure. **(B)** The silent period was shorter during the VOLT_SAS-TMS_ task compared with the VOLT_TMS_ task. ^*^Indicates statistical significant difference (*p* < 0.05).

1. MEP latency: There were no significant main effects nor interaction for MEP latency (all *p* > 0.05). Specifically, the MEP latencies were similar between the REST_TMS_ task (15.1 ± 1.24 ms), the REST_SAS-TMS_ task (15.2 ms ± 0.82 ms) task, the VOLT_SAS-TMS_ task (14.41 ± 0.95 ms), and the VOLT_TMS_ task (14.44 ± 1.72 ms).
2. MEP Amplitude: there were significant CONTRACTION [*F*_(1, 8)_ = 13.15, *p* = 0.007] and SAS [*F*_(1, 8)_ = 6.18, *p* = 0.04] main effects for MEP amplitude. Furthermore, there was a significant CONTRACTION × SAS interaction [*F*_(1, 8)_ = 7.96, *p* = 0.02]. Overall, application of SAS 50 ms prior to the TMS delivery significantly decreased the amplitude of MEP at rest (Figure 3A). *Post-hoc* analyses revealed that the MEP amplitude was smaller (*p* = 0.01) during the REST_SAS-TMS_ task (0.53 ± 0.38 mV) compared with the REST_TMS_ task (0.79 ± 0.55 mV; Figure 3B). On average, percentage of MEP reduction was about 32% at rest. In contrast, a conditioning SAS did not change the MEP amplitude during sustained voluntary contraction (Figure 4A), The MEP amplitude was similar (*p* > 0.05) between the VOLT_SAS-TMS_ task (1.37 ± 0.9 mV) and the VOLT_TMS_ task (1.32 ± 0.92 mV; Figure 4B).
3. Silent period: A conditioning SAS significantly shortened the silent period during VOLT_SAS-TMS_ task (Figure 5A). The silent period was significantly shortened (*p* = 0.03) during the VOLT_SAS-TMS_ task (187.22 ± 22.99 ms) compared with the VOLT_TMS_ task (200.56 ± 29.71 ms; Figure 5B). For both REST_SAS-TMS_ and REST_TMS_ tasks, the silent period was not identifiable.
4. TMS-induced force: Similar to the lack of change in the MEP amplitudes, the TMS-induced force was similar between the VOLT_SAS-TMS_ task (3.11 ± 2.03 N-m) and the VOLT_TMS_ task (3.62 ± 1.33 N-m).

## Discussion

In this study, TMS was delivered to the right primary motor cortex with and without a conditioning SAS to healthy subjects who were either at rest, or instructed to perform sustained left elbow flexion at 10% MVC. Our results of a significantly decreased MEP at rest with a conditioning SAS were consistent with previous findings (Furubayashi et al., 2000; Fisher et al., 2004; Kühn et al., 2004; Ilic et al., 2011). The results indicate that the transient suppression of cortical excitability suppression by SAS conditioning also happens at rest, similar to those observed during very low muscle activity. However, the effect of a conditioning SAS on cortical excitability was different during sustained voluntary contraction. A conditioning SAS resulted in a significant shortening of the MEP silent period without any change in the amplitude of MEP or TMS-induced force.

When subjects are at rest, SAS imposes a transient effect on the excitability of the primary motor cortex. It causes an early cortical inhibition about 30–60 ms after its delivery (Furubayashi et al., 2000). This effect transitions into excitation for another 50 ms with its peak aligned temporally with the event-related potential (ERP) N100 from the auditory stimuli (Löfberg et al., 2014). The early inhibition is attributed to reticulo-thalamo-cortical polysynaptic inhibition to the motor cortex (Kühn et al., 2004), while the late facilitation is related to a general cortical arousal effect (Löfberg et al., 2014). This transient effect of a SAS on the motor cortex excitability also depends on the background state of the motor system (Marinovic et al., 2014). When SAS is delivered during early preparation (2 s prior) of a reaction time task, it causes an inhibitory effect on the motor cortex. In contrast, facilitation is observed when SAS is delivered close to (0.2 s prior) the expected movement onset. At this time, the background corticospinal excitability is elevated close to its threshold for generation of voluntary movement (Li et al., 2009; Marinovic et al., 2014). The different conditioning SAS effects on the motor cortex excitability is mediated by different modulation of intracortical inhibition and facilitation due to change in background corticospinal excitability from early to late preparation readiness of a reaction time task (Marinovic et al., 2014).

In the present study, we observed no change in the MEP amplitude with a conditioning SAS during a sustained isometric contraction, in contrast to the SAS-preconditioned MEP reduction at rest. The results suggest that the conditioning SAS does not cause early inhibition on the motor cortex excitability. As compared to the rest condition, the motor cortex excitability increases and intracortical inhibition decreases during voluntary activation. Conceivably, SAS may not impose an inhibitory effect on the activated motor cortex. Similar to this study, Roshan et al. reported that a conditioning TMS pulse could suppress the test MEP at rest, but not during voluntary isometric condition at 20% MVC (Roshan et al., 2003). The authors attributed to the fact that the intracortical inhibitory circuitry is suppressed during voluntary contribution. Furthermore, results from some paired-pulse TMS protocols demonstrated that the condition effects change according to the test MEP size (equivalent to increased cortical excitability due to voluntary contraction in this study; Chen, 2004). It had been showed that a conditioning SAS reduced TMS-induced MEP during a very light contraction in previous studies (Furubayashi et al., 2000; Fisher et al., 2004; Kühn et al., 2004; Ilic et al., 2011). Different levels of voluntary contraction between previous studies and the present study might be the factor causing the conflicting results. In the present study, we measured the maximum force that the subjects could produce, and then asked the subjects to perform the 10% MVC task as precisely as they could. However, previous studies only asked the subjects to maintain a light contraction with EMG activity as the only visual feedback (Furubayashi et al., 2000) or no feedback at all (Fisher et al., 2004; Kühn et al., 2004; Ilic et al., 2011). Different levels of background activity (rest, very light, 10% MVC) may have different modulations of intracortical inhibitory circuitry, thus subsequently resulting in different effects of SAS conditioning on the MEP. A paired-pulse TMS protocol will be needed to examine this possibility.

On the other hand, no changes to the SAS-preconditioned MEP and in the TMS-induced force also indicates that SAS does not further enhance motor cortical excitability. This suggests that SAS causes a general arousal activation effect at the cortical level (Löfberg et al., 2014), while the corticospinal motor output is not altered.

The silent period is a transient suppression of motor activity after a TMS-induced muscle response during muscle contraction, whereas MEPs are a reflection of the activation of corticospinal projections (Fuhr et al., 1991). Although the mechanisms of the silent period are not well understood, it is generally accepted that the initial segment is of spinal and later part of cortical origin (Fuhr et al., 1991; Cantello et al., 1992; Chen et al., 1999). The duration of the silent period depends primarily on the intensity of TMS. It does not correlate to the level of muscle activation (Säisänen et al., 2008). With the same intensity of TMS, we observed a significant shortening of the silent period by the conditioning SAS. This shortening of the silent period is likely mediated by SAS-activated reticulospinal projections. As an indicator of spinal motor neuron excitability, the H-reflex amplitude is increased by a conditioning SAS, concomitant with MEP suppression (Ilic et al., 2011).

Collectively, our results demonstrate that a conditioning SAS does not cause a transient inhibitory effect on the motor cortex during voluntary activation, in contrast to the SAS-preconditioned cortical suppression at rest. The result of a significant shortening of the silent period suggests that the conditioning SAS has a separate facilitatory effect on spinal motor neurons via activation of descending reticulospinal projections during sustained voluntary contraction. In this study, the SAS-related reticulospinal facilitation seems to not contribute to the force output, as we did not observe a significant increase in TMS-induced force. This could be related to the fact that only a low level of voluntary contraction was tested. Reticulospinal contributions to force output may be manifested if higher levels of sustained contraction were tested. It has been recently shown that reticulospinal projections may contribute to sustained voluntary contraction in stroke survivors when the level of activation progressively increases (Chang et al., 2013). Furthermore, in spastic hemiplegic stroke survivors with elevated reticulospinal excitability, SAS-related reticulospinal activation may contribute to force output at low levels of sustained activation (Bhadane et al., 2015). Further research on this is needed. It may expand clinical application of auditory cueing for initiation (Whitall et al., 2000; Schneider et al., 2007; Jun et al., 2013; Pollock et al., 2014) to a SAS-integrated strengthening program for stroke rehabilitation.

## Conclusion

In summary, we confirm a transient SAS-preconditioned cortical suppression at rest in this study. Our results further demonstrate that there is no SAS-preconditioned MEP suppression, but a significant shortening of the silent period during sustained voluntary contraction.

## Author contributions

Experimental design: YC, SAL, PZ, and SL. Data Collection: YC, SAL, SL. Data Analysis and interpretation: YC, SAL, PZ, and SL. Manuscript Draft: YC and SL. Discussion and final approval: YC, SAL, PZ, and SL.

### Conflict of interest statement

The authors declare that the research was conducted in the absence of any commercial or financial relationships that could be construed as a potential conflict of interest.

## Abbreviations

SAS: startling acoustic stimuli
TMS: transcranial magnetic stimulus
MEP: motor evoked potential
ASR: Acoustic startle reflex
SES: subcortical electrical stimulation
RESTTMS: TMS at rest without SAS
RESTSAS-TMS: TMS at rest with a conditioning SAS
VOLTTMS: TMS during voluntary muscle contractions without SAS
VOLTSAS-TMS: TMS during voluntary muscle contractions with a conditioning SAS.
